# Tunable White Light for Elders (TWLITE): A Protocol Demonstrating Feasibility and Acceptability for Deployment, Remote Data Collection, and Analysis of a Home-Based Lighting Intervention in Older Adults

**DOI:** 10.3390/s22145372

**Published:** 2022-07-19

**Authors:** Jonathan E. Elliott, Carolyn E. Tinsley, Christina Reynolds, Randall J. Olson, Kristianna B. Weymann, Wan-Tai M. Au-Yeung, Andrea Wilkerson, Jeffrey A. Kaye, Miranda M. Lim

**Affiliations:** 1 VA Portland Health Care System, Research Service, Portland, OR 97239, USA; elliojon@ohsu.edu (J.E.E.); jocaro@ohsu.edu (C.E.T.); olsonra@ohsu.edu (R.J.O.); 2 Department of Neurology, Oregon Health & Science University, Portland, OR 97239, USA; dunch@ohsu.edu (C.R.); auyeungm@ohsu.edu (W.-T.M.A.-Y.); kaye@ohsu.edu (J.A.K.); 3 Department of Behavioral Neuroscience, Oregon Health & Science University, Portland, OR 97239, USA; 4 School of Nursing, Oregon Health & Science University, Portland, OR 97239, USA; weymannk@ohsu.edu; 5 Pacific Northwest National Laboratory, Portland, OR 97239, USA; andrea.wilkerson@pnnl.gov; 6 Oregon Institute of Occupational Health Sciences, Oregon Health & Science University, Portland, OR 97239, USA; 7 Pulmonary and Critical Care Medicine, Oregon Health & Science University, Portland, OR 97239, USA; 8 VA Portland Health Care System, Mental Illness Research Education and Clinical Center, Neurology, National Center for Rehabilitative Auditory Research, Portland, OR 97239, USA

**Keywords:** protocol, tunable light, sleep, Alzheimer’s, smart living applications

## Abstract

Sleep disturbances are common in older adults and may contribute to disease progression in certain populations (e.g., Alzheimer’s disease). Light therapy is a simple and cost-effective intervention to improve sleep. Primary barriers to light therapy are: (1) poor acceptability of the use of devices, and (2) inflexibility of current devices to deliver beyond a fixed light spectrum and throughout the entirety of the day. However, dynamic, tunable lighting integrated into the native home lighting system can potentially overcome these limitations. Herein, we describe our protocol to implement a whole-home tunable lighting system installed throughout the homes of healthy older adults already enrolled in an existing study with embedded home assessment platforms (Oregon Center for Aging & Technology—ORCATECH). Within ORCATECH, continuous data on room location, activity, sleep, and general health parameters are collected at a minute-to-minute resolution over years of participation. This single-arm longitudinal protocol collected participants’ light usage in addition to ORCATECH outcome measures over a several month period before and after light installation. The protocol was implemented with four subjects living in three ORCATECH homes. Technical/usability challenges and feasibility/acceptability outcomes were explored. The successful implementation of our protocol supports the feasibility of implementing and integrating tunable whole-home lighting systems into an automated home-based assessment platform for continuous data collection of outcome variables, including long-term sleep measures. Challenges and iterative approaches are discussed. This protocol will inform the implementation of future clinical intervention trials using light therapy in patients at risk for developing Alzheimer’s disease and related conditions.

## 1. Introduction

Sleep disturbances are common in older adults and feature prominently in individuals with Alzheimer’s disease (AD), affecting between 25% and 60% of all patients [1,2,3,4]. Sleep disturbances, including insomnia, sleep fragmentation, and excessive daytime sleepiness, may even precede the onset of cognitive symptoms in patients with AD [5,6,7]. In addition to sleep complaints, AD is also associated with disturbances in the circadian rhythm, which likely contributes to “sundowning” in later stages of disease [5,8]. Furthermore, sleep itself is critical for normal memory function and consolidation; poor sleep causes deficits in synaptic plasticity and memory [9,10,11], which likely also contribute to the decline in cognition and memory in AD. Recent evidence from animal and human studies suggests that lack of sleep increases the levels of soluble amyloid-beta (Aβ) and tau in the brain, in turn, exacerbating AD plaque and tangle pathology [12,13]. The mechanisms underlying the impact of sleep on AD pathology are not well understood but could include decreased clearance of soluble Aβ and tau that is normally facilitated during sleep [14,15]. Thus, improving sleep may also improve pathological and clinical outcomes in AD, potentially through improved/restored clearance of soluble Aβ and tau.

However, current pharmacological and non-pharmacological therapies for sleep disturbances in AD are limited. Sleep hygiene practices, including limiting caffeine and alcohol intake, avoiding evening light exposure from computers or the television, exercising regularly, and keeping regular bedtimes and waketimes with adequate light exposure upon waking, are a start but limited in efficacy and adherence [16,17]. Adequate daytime light exposure is a critical issue in older-aged populations, especially in institutionalized individuals. For example, recent measurements at two senior care facilities in the US with fluorescent lighting systems found the average vertical illuminance at 1.25 m above the floor ranged from 111 to 128 lux in the hallways, over 350 lux in the dining rooms, and 85 to 386 lux in the activity rooms [18]. In another study of aged care residents, the median daytime light exposure was only 52 lux [19]. Retinal light exposure has a myriad of biological functions, including being a central zeitgeber for mammals [20] primarily responsible for entraining endogenous circadian rhythms. Circadian entrainment occurs via the circadian pacemaker located in the hypothalamic suprachiasmatic nuclei (SCN), responsible for communicating with other brain regions to synchronize physiologic and behavioral functions [21,22]. Aging is associated with impaired circadian rhythms, potentially due to fewer SCN cell numbers and/or reduced activity, demonstrated via a lower oscillation amplitude in response to light [23]. However, it is also possible that aging is associated with inactive SCN cells that can be reversed with exposure to bright light, as has been shown in rat models of aging [24]. Additionally, aging is associated with a variety of other factors that can contribute to impaired circadian rhythms, including physical immobility-related restrictions to natural light [25], and ocular issues such as yellowing of the lens [26] and a reduced pupil diameter [27] and increased sensitivity to glare. All combined, people over 75 years of age retain ~20% of a 10-year-old child’s photoreceptor capacity and, therefore, require brighter light and/or exposures of longer duration to maintain optimal circadian rhythms [27]. Thus, more regular light exposure could help better entrain dysfunctional circadian rhythms in AD, and possibly improve physical activity during the day [28]. However, studies have differed in the delivery, timing, dosage, and duration of light therapy, with variable contributions from specific wavelengths, and variable outcomes, including sleep, circadian realignment, cognition, and mood [29].

Bright light therapy likely exerts its pleiotropic effects through activation of intrinsically photosensitive retinal ganglion cells (ipRGCs), the starting point of the non-image-forming pathway. ipRGCs are maximally sensitive to light in the shorter wavelengths (e.g., blue light, λ = 460–480 nm) and less so to longer wavelengths, including green, amber, and red [30,31,32]. Bright white (e.g., broad spectrum) light effectively alters circadian rhythms, particularly the timing of melatonin release [33,34]. A meta-analysis by van Maanen et al. in 2016 [34] reported a significant benefit of light therapy for sleep disturbances in dementia, sleep onset latency, total sleep time, time in bed, and sleep efficiency [35]. Recent work by our group has shown a beneficial effect of light therapy isolated to the morning hours on sleep in older adults with a history of brain injury [36]. Other work has broadened this to examining blue-enriched light installed as overhead fluorescent lighting within care homes of older subjects (>65 years of age) and found that round-the-clock blue-enriched lighting improved daytime alertness and anxiety but, unsurprisingly, also reduced sleep at night [31]. In contrast, a tunable lighting system installed in the hallways of a 99-bed care center reduced sleep disturbances by 50% compared to static lighting [37]. Other recent work has explored bright light therapy (1 h/day; 2 weeks) specifically in AD and patients with dementia with Lewy bodies (DLB) and found sleep was improved in AD but not DLB [38]. This study, while promising, may have been improved had the lighting intervention been tuned to maximize blue light in the morning and minimize blue light in the evening. Indeed, the value of an automated tunable white light solution has been previously reviewed [39]. Supporting the value of this approach is a recent feasibility and acceptability report of two case studies in individuals post-traumatic brain injury with a whole home tunable lighting system installed [40].

In summary, sleep disturbances are not only common in old age but critically impact the pathogenesis of AD and other conditions. Light therapy is a promising low-risk intervention to improve sleep in these populations, but to date, limited work has been carried out in this field, specifically to develop a dynamic protocol that maximizes both daytime and nighttime effects. Herein, we describe our protocol to implement a whole-home tunable lighting system and determined the feasibility and participant acceptability of this system as a potential sleep and health intervention. This protocol (TWLITE: Tunable White LIghT for Elders) will inform future full-scale randomized controlled trials using this approach.

## 2. Materials and Methods

**Figure 1:**
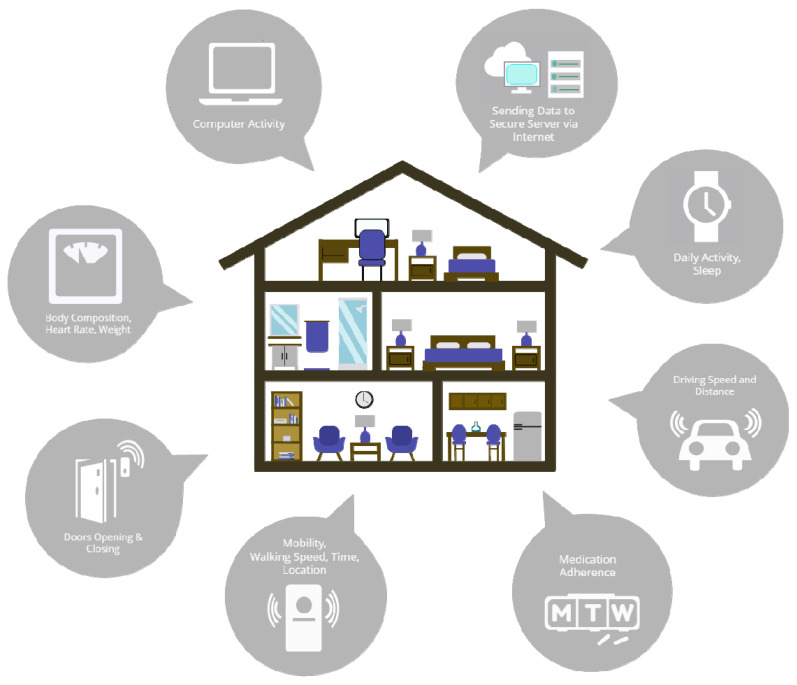
Illustration of the full suite of integrated assessment platforms built into participants’ homes (Life Lab), through The ORegon Center for Aging and TECHnology (ORCATECH) supported by the NIH National Institute of Aging as a designated Roybal Center.

The VA Portland Health Care System approved this project, and each subject gave written and verbal informed consent prior to participation (IRB #4447). This pilot project included installing tunable white LED-based lights in 3 homes beginning in early 2020, one of which was a dyad and, therefore, involved a total of *n* = 4 participants. The lighting system in one of the three homes was not functional and due to the COVID-19 pandemic-associated lockdown in Oregon, study personnel were not allowed into the home to troubleshoot. The remaining two homes remained fully operational for the next 18 months. Therefore, outcome metrics are reported for these two homes (*n* = 3 participants). This single-arm open-label longitudinal design enrolled subjects who were part of The ORegon Center for Aging and TECHnology (ORCATECH), all with existing home assessment platforms allowing continuous data collection of various outcomes (Life Lab; Figure 1). We present an additional up to 12 months of relevant data (e.g., passive infrared motion sensor firing, sleep metrics, and wrist-based actigraphy) from this in-home assessment platform prior to light installation. All data, code, and protocol design are publicly available upon request.

### 2.1. Lighting Protocol

The tunable white light system deployed was based off the Philips Hue lighting system (Signify N.V., brand name Philips Lighting, Eindhoven, The Netherlands). The light bulbs installed were Philips Hue White Ambiance with an E26 fitting and A19 form factor. These bulbs communicate with the proprietary Philips Hue Bridge (each bridge supports a maximum of 50 light bulbs) via local Wi-Fi, which can functionally be controlled via the Philips Hue App. However, the standard user-facing app did not allow for the level of control needed and required bypassing the app to communicate directly with the Hue Bridge. This was accomplished by using an onboard Linux microcomputer (Raspberry Pi; v. 4, Cambridge, UK) to communicate via the Hue Bridge Application Programming Interface (API). The Raspberry Pi control unit also enabled local storage of data and the ability to remotely access these data, and the flexibility to either use Wi-Fi or wired ethernet connection. The Linux-based Raspberry Pi terminal runs a custom bash script, along with a Hue bash library to facilitate communication with the Hue API. Operationalizing this requires (1) powering on all Philips Hue light bulbs, (2) using the Hue App to associate all bulbs with the Hue Bridge, (3) switching full control to the Raspberry Pi to bypass the Hue App, and (4) running the custom bash scripts.

Every day at 12:00 A.M., the bash script executed, which calculated the duration between sunrise and sunset (in seconds), both of which were determined based on the location and date. The spectrum of the light emitted was automatically adjusted throughout the day between micro-reciprocal degrees, i.e., mired value, of 153 (i.e., cool light; 6500 K) to a mired value of 454 (i.e., warm light; 2200 K) at a constant rate depending on the duration of time between sunrise and sunset. The light output began shifting from warm to cool starting at 04:00 h and gradually increased to a mired value of 153 at sunrise. This level was maintained until 12:00 h, at which point the light output gradually warmed until sunset when it reached a mired value of 454. The light output between sunset at 04:00 remained at a constant mired value of 454 in case participants needed to turn lights on. As will be discussed further, this tunable lighting schedule thus mirrored participants’ natural environment and the same spectrum of light they would be exposed to if outdoors.

### 2.2. Passive Infrared Motion Sensors

In-home passive infrared (PIR) motion sensors (NYCE Sensors; Burnaby, BC, Canada) were affixed to the wall in four common rooms within the homes: bathroom, bedroom, kitchen, and living room as previously described [41,42,43]. Each room and door were assigned unique identifiers, and thus the firings of sensors in rooms recorded the presence of a participant within a specific room or entrance. Continuous sensor firings for each room were compiled and plotted daily over the 12-month duration of the study.

### 2.3. Sleep

The determination of participants’ bedtimes and waketimes was achieved through the installation of Emfit bedmats (Emfit Corp., San Marcos, TX, USA), previously validated against wrist-based actigraphy [44], while leveraging the existing PIR motion sensor network. Of note, due to the COVID-19 pandemic and associated lockdown in Oregon, we were unable to access participants’ homes for the installation of bedmats until 3 months after the light installation. Accordingly, the sleep estimation algorithm was created using a combination of PIR motion sensor firing data, with cross-validation via Emfit bedmat mattress sensor data (developed by Wan-Tai M. Au-Yeung Oregon Health & Science University).

The algorithm steps were as follows: First, all PIR sensors in the home were verified to be fully functioning. If any of these motion sensors were determined to be temporarily non-functioning (e.g., experiencing a glitch, network disconnection, etc.), then the algorithm was terminated and would not provide an output. Once it was determined that all PIR motion sensors were fully functional and were detecting an individual within the home, then, the sleep onset time and wake time were determined as follows: (1) mark the period of time the participant was inside the home during the night (e.g., identify the longest period of time between two door state changes between 6 pm on the first day and 11 am on the second day); (2) identify all sedentary periods when no motion was detected for 20 min or longer; (3) label the sleep onset time by finding the start time of the first three consecutive sedentary periods between which there were no sustained activities longer than 20 min; and (4) label the wake time by finding the end time of the last sedentary period when the participant was in home before his/her first exit in the morning—if this wake time was between 9 am and 11 am, we would determine whether there were any sustained activities longer than 15 min before that. If this was the case, then the wake time would be changed to the time just prior to the start of these sustained activities.

### 2.4. Actigraphy

Daily activity, determined via the number of total steps, was collected using the Withings Activite actiwatch (Withings; Issy-les-Moulineaux, France). This actiwatch has been used within the ORCATECH home assessment platform and specifically for studies related to the national Collaborative Aging Research using Technology (CART) initiative [45] and the Ecologically Valid Ambient Longitudinal and Unbiased Assessment of Treatment Efficacy in Alzheimer’s Disease (EVALUATE-AD) study [46]. The Withings Activite has previously been validated against the Actigraph wGT3X-BT (Pensacola, FL, USA) and StepsCount PiezoRxD (Deep River, ON, Canada) [47].

## 3. Results

### 3.1. Demographics

Home 1422 included one male participant, and home 1286 included one male and one female participant. All subjects were non-demented and between 75 and 89 years of age. The ORCATECH Life Lab technology platform has an extensive array of sensors already installed throughout each home (Figure 1), the data from which is all automatically sent to a secure data server. This includes 24/7 wrist-based actigraphy for activity tracking, sensors within door frames tracking opening/closing, infrared motion sensors enabling the tracking of walking speed and location, smart scales monitoring body composition/weight and heart rate, computer activity tracking (e.g., time on computer), medication adherence via electronic pill boxes, and GPS tracking within participants’ cars providing data related to driving metrics (e.g., braking, speed) and distance traveled. The presentation of the full suite of available data is beyond the scope of this manuscript. Here we show in addition to the light installation protocol, data related to the PIR, bedmat, and activity tracker.

### 3.2. Feasibility and Acceptability

One subject terminated the study, citing technical difficulties with the lights and a preference for brighter illumination. Due to restrictions on human subject research related to the COVID-19 pandemic, research staff were not able to enter the homes to fix these issues. The remaining two homes (three participants) reported no technical difficulties and were highly agreeable to the overall protocol (i.e., change in spectrum). Specific program successes and challenges are outlined in Table 1. We demonstrated a functioning automated program to control light color temperature (spectrum) following seasonal changes in length of day, time of sunrise/sunset, and otherwise optimized to human biological function. Challenges for research investigators included that it was not possible to instantaneously verify whether each light was functioning correctly nor confirm that the light exposure received was the appropriate wavelength. We propose as a solution to this issue: to incorporate an in-home sensor with integrated closed loop functionality. Full implementation of this system necessitates relatively extensive installation and, therefore, at this point, it will require an appropriately skilled research or clinical staff member to perform. This is particularly salient if considering the installation of this system in a population with cognitive impairment.

### 3.3. Home Floorplans

**Figure 2:**
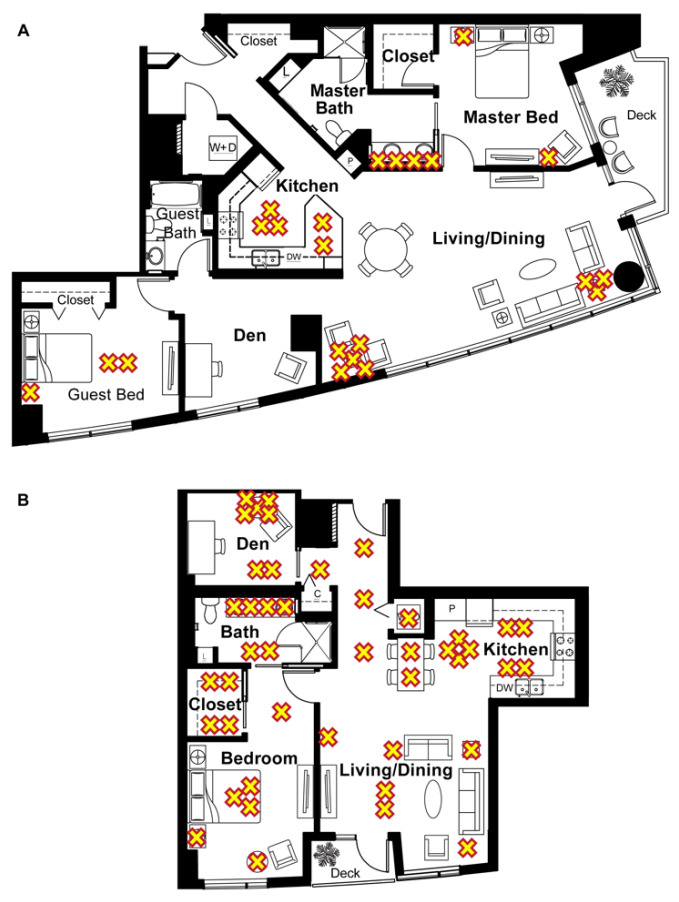
Floor plans of the homes included in the study and the approximate location of the light fixtures, indicated by a yellow “X”. (**A**) Floor plan of home 1286, a two-bedroom unit with *n* = 2 subjects and *n* = 22 lights. (**B**) Floor plan of home 1422, a one-bedroom unit with *n* = 1 subject and *n* = 43 lights.

Home 1286 (Figure 2A) and home 1422 (Figure 2B) were located in Portland, OR, USA. Home 1286 had two bedrooms and ~150 m^2^ of living space while home 1422 had one bedroom and ~93 m^2^ of living space. The approximate locations of the lights are identified by yellow Xs. Home 1286 had 22 light bulbs installed and home 1422 had 43 light bulbs installed.

### 3.4. Mired Values and Light State

**Figure 3:**
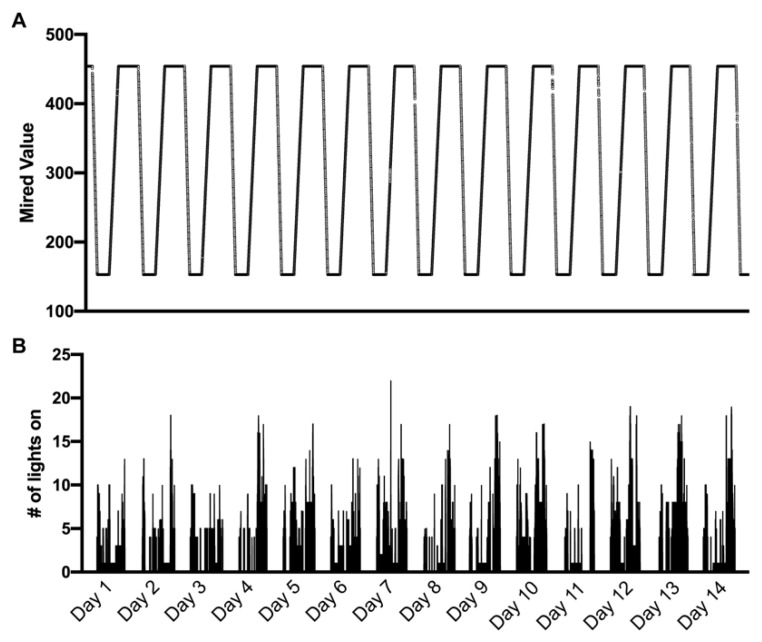
Mired values from home 1286 showing the programmed cycle of light spectral changes over the course of 24 h between February and March 2020 (**A**), with the number of light fixtures turned on (maximum of 22) at a 1-s resolution corresponding to the same timespan between February and March 2020 (**B**). This shorter timescale improves visualization within panel B; however, these data were collected continuously.

Mired values pulled from home 1286 showing the programmed cycle of light spectral changes over the course of 24 h are presented in Figure 3A between February and March for 14 consecutive days. The temperature of light produced was automatically adjusted throughout the day between a mired value of 153 (cool light; 6500 K) to a mired value of 454 (warm light; 2200 K) at a constant rate depending on the duration of time between sunrise and sunset. The light output began shifting from warm to cool starting at 04:00 h and gradually increased to a mired value of 153 at sunrise. This level was maintained until 12:00 h, at which point the light output gradually warmed until sunset when it reached a mired value of 454. The light output between sunset at 04:00 remained at a constant mired value of 454 in case participants needed to turn lights on. For illustration purposes, we show one light, highlighting small discrepancies in the light output and adherence to the script. The remaining 21 lights followed a highly comparable pattern; however, overlaying all 22 lights obstructs a clear view of the stepwise increase and relevant, albeit minor, discrepancies.

The number of light bulbs turned on was summed at a frequency of ~1 Hz and plotted relative to the same 14 consecutive day timespan (Figure 3B).

### 3.5. Passive Infrared Motion Sensors

**Figure 4:**
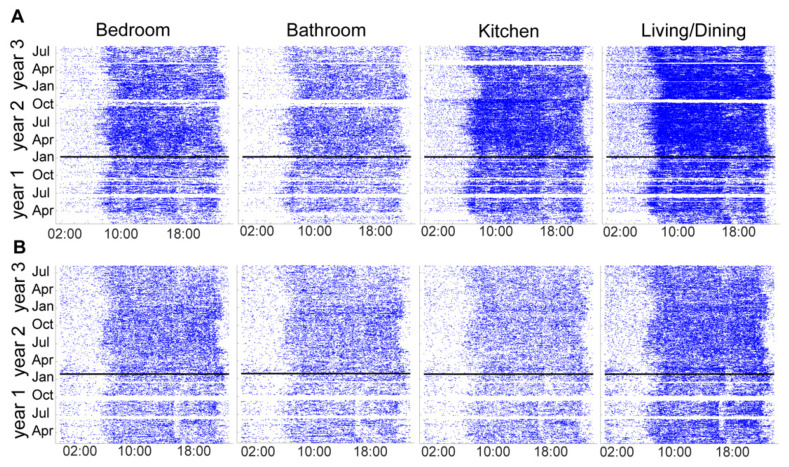
Passive infrared (PIR) motion sensors located in participants’ main living spaces (primary bedroom, primary bathroom, kitchen, and living/dining room) illustrating movement within these rooms across time. (**A**) Room sensor firings in home 1286. (**B**) Room sensor firings in home 1422. The horizontal line indicates when the lights were installed in each home.

The PIR motion sensor firings from four rooms (primary bedroom, primary bathroom, kitchen, and living/dining room) in both homes from March of year 1 until July of year 3 are shown in Figure 4A for home 1286 and Figure 4B for home 1422. The dates of the light installation in the respective homes are represented by a horizontal black line.

### 3.6. Sleep

**Figure 5:**
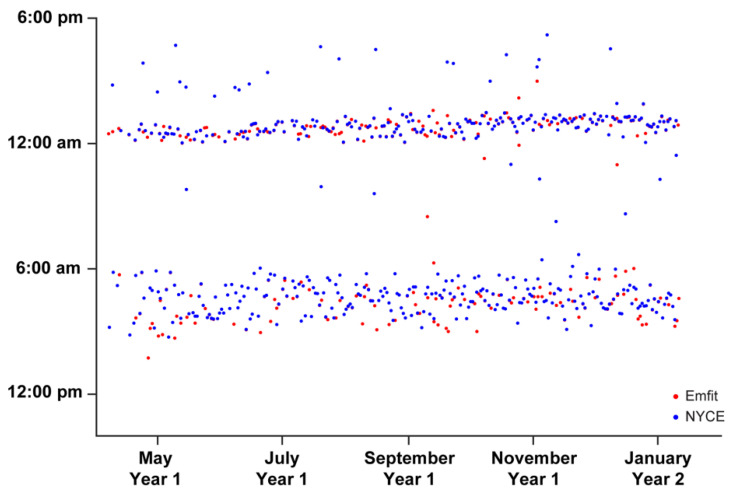
Participant’s bedtimes (evening hours) and waketimes (morning hours) recorded via the Emfit bedmat and passive infrared (PIR) motion sensors network. The PIR sleep estimation algorithm was created using a combination of PIR motion sensor firing data, with cross-validation via Emfit bedmat mattress sensor data (developed by W.-T.M.A.-Y.). This figure illustrates *n* = 1 participant.

Bedtimes and waketimes recorded by the Emfit bedmat and the PIR motion sensor-related algorithm are plotted in Figure 5. Here, we show agreement between these two metrics, with the primary outliers coming from the PIR motion sensor algorithm. The most likely explanation for these data reflects participants falling asleep in a chair or couch (e.g., while watching television) prior to getting into bed.

### 3.7. Physical Activity (Steps)

**Figure 6:**
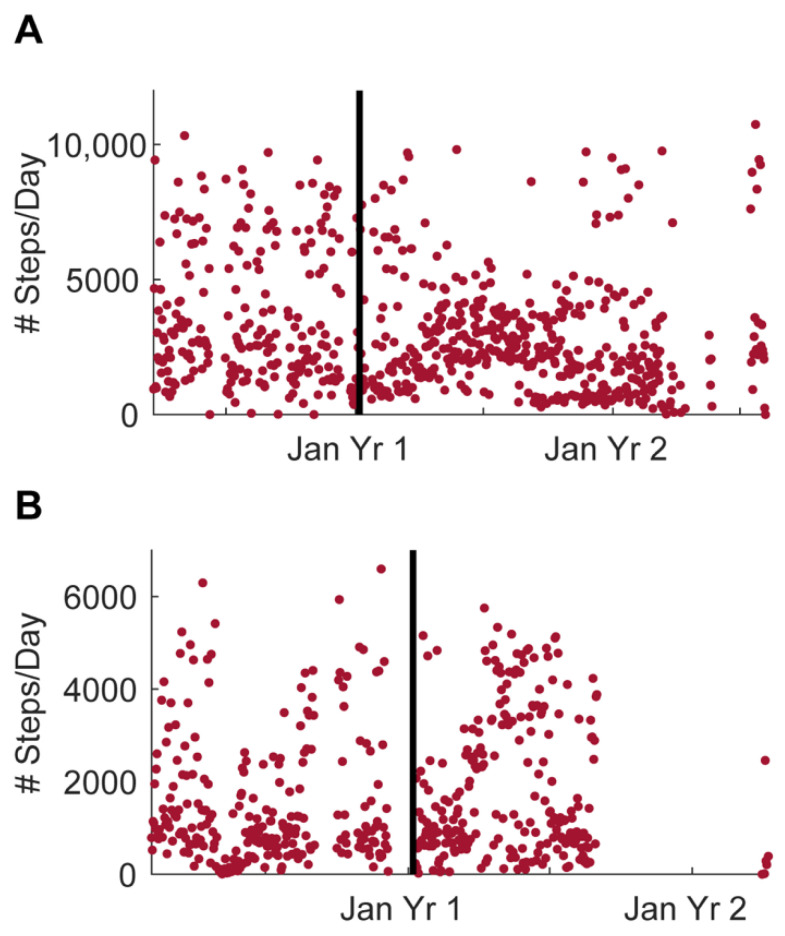
Daily steps from one participant in each home collected using the Withings Activite actiwatch. (**A**) Daily steps from one participant in home 1286. (**B**) Daily steps from the only participant in home 1422. The horizontal line indicates when the lights were installed in each home.

The number of steps from one participant in home 1286 is shown in Figure 6A and the single resident of home 1422 Figure 6B from March 2019 until July 2021.

## 4. Discussion

In this report, we present a protocol to install dynamic, automated tunable lighting integrated into native home lighting systems as a potential sleep intervention for an older population at risk for Alzheimer’s disease (AD) and related dementia. This protocol, TWLITE (Tunable White LIghtT for Elders), presents feasibility and acceptability outcomes in support for implementation in full-scale randomized clinical trials. We integrated the tunable lighting system with an existing open-source home assessment platform (ORCATECH; ORegon Center for Aging and TECHnology) to allow continuous data collection of digital biomarkers. These digital biomarkers included room location, activity, and long-term estimated sleep measures. Overall, the protocol was feasible, with three of four subjects participating in the study for over 12 months. Reasons for dropout and barriers to full acceptance were primarily related to the inability of staff to enter homes during the COVID-19 pandemic. In summary, this pivotal feasibility protocol will inform the implementation of future clinical intervention trials using customizable light therapy in patients at risk for developing AD.

These data support the premise that tunable whole-home lighting systems are highly acceptable and feasibly implemented using an automated platform for continuous data collection, especially in an older population. This older population may present more challenges for the use of in-home technology, for a variety of reasons not limited to cognitive impairment and resistance to change in routine. Therefore, it is critical that any potential interventions be as turnkey and automated as possible. At present, this protocol requires skilled installation; however, future iterations will strive to improve the user installation experience. Interventions integrated within the home, rather than requiring visits outside the home, will ensure adherence without requiring any changes to normal home routines. However, seamless integration within the home environment has its challenges as all homes are different. We observed a wide range of variations in the home lighting environment. Not only did homes differ widely in the number of light fixtures (dependent, in part, on the window area within rooms) but there was also large variation in the types of lights (e.g., sockets, orientation, diffusors, etc.). Any future study preparing home installation of lights will need to take these factors into consideration.

In addition to the feasibility of implementing this protocol, our results show that the collection of activity and sleep over long periods of time is robust and reliable in the home environment of older subjects.

Exploratory analysis of data from this study allowed us to develop a novel sleep estimation algorithm to cross-validate data from PIR sensors and bedmats from the same individuals. This algorithm is able to estimate sleep over the long term using PIR sensor firing data alone, a digital biomarker in place within over 400 homes worldwide.

While our pilot study was not designed to examine the efficacy of the TWLITE (Tunable White LIghtT for Elders) intervention on sleep, it illustrates the breadth and depth of data that could be collected long term in a future intervention trial. Planned outcome variables, such as room location, room transitions (as in [42]), bedtimes, waketimes, sleep duration, and steps, yield a comprehensive dataset that paints an integrated picture of an individual’s whole health. Additional outcome variables may also be explored with this dataset and protocol, which may be associated with cognitive decline. For example, increased room transitions and dampened infradian rhythms in the amplitude of sleep duration across seasons have both been found to be worse in persons with mild cognitive impairment [42,48]. Indeed, the PIR sensor firing data in Figure 4 provide clear graphic visualization of the seasonal effects on activity and sleep duration. It is possible that environmentally mediated variables may respond to a lighting intervention.

Herein, we also describe a novel algorithm with which to estimate sleep in participants who have both PIR sensors and bedmat data. The addition of the bedmat is a relatively recent development in the ORCATECH platform that has not been previously published in this context. Recent work by our group has validated the bedmat against wrist actigraphy in healthy young human subjects [44]. However, the bedmat has not yet been validated against the PIR motion sensors. Our algorithm estimates sleep from room location activity using the bedmat as the gold standard and appears to be highly accurate in this limited dataset. However, outliers do appear, and are likely due to prolonged inactivity prior to bedtime (e.g., watching television in the living room). One approach to outliers is to fit a normal distribution to the sleep onset times and waketimes separately and exclude values if they are more than two to three standard deviations away from the mean. If there is substantial data such that the impact of occasional missingness is minimal, this method of outlier exclusion would be preferable to overfitting the algorithm to the data from a single home, as participant behavior likely shows substantial individual variation. A final caveat is that this sleep estimation algorithm is only valid in homes occupied by single dwellers since the PIR motion sensors cannot distinguish more than one individual.

Finally, our protocol has its limitations. First, a fully remote deployment may not be possible given our target population may be less technologically savvy and have cognitive impairment. Therefore, technicians will need to be willing and able to enter the home to assess the lighting fixtures and connectors and installation of the Philips Hue equipment and Raspberry Pi controller device. Secondly, one major variable that is missing from the assessment platform is a high-resolution light sensor that can detect and record multiple wavelengths of light. While commercially available devices exist to detect and record illuminance levels (e.g., HOBO, Omega Engineering Inc., Norwalk, CT, USA), none yet record the spectral power distribution of the light in a small form factor that can be readily integrated into the ORCATECH platform or other whole home systems. There are spectrometers in development that may soon meet the above criteria, and these devices will be critical to confirming participants are receiving the intended spectral light exposure. Third, it remains unknown what exposure level (i.e., lux) of light participants experienced. Fourth, the present report is “single blind”, which, while appropriate for the intended feasibility and acceptability focus of this protocol report, would be improved if double-blind/controlled in future full-scale studies. Possible approaches to a control system would be to fix the light spectrum at a standard commercial output compared to the tunable dynamic schedule in a cross-over-based design such that each participant is their own control. In summary, these results provide valuable information in the design and implementation of future large-scale lighting intervention studies in patients at risk for developing AD.

## Figures and Tables

**Table 1:** Feasibility and participant acceptability.

**Successes**
Automated program to control color temperature worked well
Subjects could continue to use native home light switches
Combination of Raspberry Pi & Philips Hue system allowed collection of light usage data
**Challenges**
Lights too dim (*n* = 1 subject)
Color temperature preference of subjects not accounted for
Custom converters required for wide variety of sockets/lamp types
No way to instantaneously check if lights are working
No way to confirm light exposure received (solution is to incorporate a closed loop light sensory)

## Data Availability

A de-identified, anonymized dataset will be created and shared, and released once institutional approvals are in place. Where practicable, sharing will take place under a written agreement prohibiting the recipient from identifying or re-identifying (or taking steps to identify or re-identify) any individual whose data are included in the dataset. However, it is permissible for final datasets in machine-readable format to be submitted to and accessed from PubMed Central (and similar sites) provided that care is taken to ensure that the individuals cannot be re-identified using other publicly available information. De-identified data sets will be maintained locally on VA computer drives that are regularly backed up and password protected until central data repositories become available for long-term storage and access. Until that time, the data sets will be available by request to any interested investigators together with a data dictionary to enable interpretation of the data set.
